# Phylogenetic Relationships of Tribes Within Harpalinae (Coleoptera: Carabidae) as Inferred from 28S Ribosomal DNA and the *Wingless* Gene

**DOI:** 10.1673/031.008.6301

**Published:** 2008-10-23

**Authors:** Karen A. Ober, David R. Maddison

**Affiliations:** ^1^ Interdisciplinary Program in Insect Science, University of Arizona, Tucson, Arizona 85721, U.S.A; ^2^ Department of Biology, College of the Holy Cross, Worcester, Massachusetts 01610, U.S.A; ^3^ Department of Entomology, University of Arizona, Tucson, Arizona 85721, U.S.A; ^4^ Present address: Department of Biology, College of the Holy Cross, Worcester, Massachusetts 01610

**Keywords:** beetle systematics, ground beetles, large subunit ribosomal DNA, lebiomorph, nuclear genes, wingless

## Abstract

Harpalinae is a large, monophyletic subfamily of carabid ground beetles containing more than 19,000 species in approximately 40 tribes. The higher level phylogenetic relationships within harpalines were investigated based on nucleotide data from two nuclear genes, *wingless* and 28S rDNA. Phylogenetic analyses of combined data indicate that many harpaline tribes are monophyletic, however the reconstructed trees showed little support for deeper nodes. In addition, our results suggest that the Lebiomorph Assemblage (tribes Lebiini, Cyclosomini, Graphipterini, Perigonini, Odacanthini, Lachnophorini, Pentagonicini, Catapiesini and Calophaenini), which is united by a morphological synapomorphy, is not monophyletic, and the tribe Lebiini is paraphyletic with respect to members of Cyclosomini. Two unexpected clades of tribes were supported: the Zuphiitae, comprised of Anthiini, Zuphiini, Helluonini, Dryptini, Galeritini, and Physocrotaphini; and a clade comprised of Orthogoniini, Pseudomorphini, and Graphipterini. The data presented in this study represent a dense sample of taxa to examine the molecular phylogeny of Harpalinae and provide a useful framework to examine the origin and evolution of morphological and ecological diversity in this group.

## Introduction

The carabid beetles are one of the largest groups of terrestrial predators on earth with nearly 35,000 species worldwide (Lorenz 2005). Most of the carabid species are found within the subfamily Harpalinae, a large, well-defined clade that has diversified into over 19,000 species (Lorenz 2005) since the Cretaceous period (Lindroth 1974; Ponomareko 1989; 1992). The monophyly of Harpalinae is well established from 18S rDNA, 28S rDNA, and the *wingless* (*wg*) gene (Maddison et al. 1999; Ober 2002), as well as morphological characteristics such as loss of the seta in the scrobe of the mandible, loss of setae from the male parameres, and marked asymmetry of the parameres (Jeannel 1941). In addition, weaker evidence is provided by the fact that most harpalines use formic acid and hydrocarbons in their defensive secretions (Kanehisa and Kawazu 1985; Kanehisa and Murase 1977; Moore 1979; Moore and Wallbank 1968), and most have a chromosome number in males of 2N = 36 + X (Serrano 1992; Serrano and Yadav 1984).

Harpalines live in a diversity of habitats including deep inside caves (Liebherr and Samuelson 1992; Moore 1995; Ortuno and Salgado 2000) and at the top of tropical rainforest canopies (Erwin 1979a). Some harpalines exhibit unusual morphological forms such as an elongated body in the genus *Agra*, an ant-like form in *Calybe*, snail-shell cracking mandibles in *Licinus* (Brandmayr and Brandmayr 1986), and an extremely dorso-ventrally flattened body as in *Mormolyce*. Not only are members of Harpalinae diverse in morphological form, but they display a variety of unusual lifestyles including granivory and herbivory (Forsythe 1982; Lindroth 1968), myrmecophily and termitophily (Brandmayr et al. 1994; Erwin 1981), ovoviparity (Liebherr and Kavanaugh 1985), ectoparasitism of other insects (Jolivet 1967; Lindroth 1954), specialized host mimicry by ectoparasites and sequestering toxins from their hosts for their own defense (Balsbaugh 1967; Lindroth 1971), and predation of vertebrates such as frogs (Elron et al. 2007).

Exploration of evolution of behaviors including specialized prey feeding and ovoviparity, close association with other organisms such as ants and termites, and specialized habitat preferences such as arboreality and cave-dwelling, and morphological characters associated with these habitats, as well as biogeographical patterns has been hindered by the lack of a well-supported phylogenetic hypothesis of the relationships within Harpalinae. A phylogeny is also critical in understanding the evolution and diversity of tribes, genera, and species within harpalines.

The history of carabid systematics has involved the progressive integration of various morphological (Beutel and Haas 1996; Liebherr and Will 1998; among others) and molecular characters (Maddison et al. 1999), however, the relationships of harpaline tribes have been unstable. Previous studies (Arndt 1993; Ball 1979; Erwin 1985; 1991a; 1991b; Jeannel 1941; Kryzhanovsky 1976) suggested monophyletic groups and sister group relationships based on similarities in one or a few morphological character systems, but phylogenetic analyses was not used to test these relationships. Maddison et al. (1999) were the first to apply molecular tools to study the phylogenetic relationships within Carabidae using 18S rDNA sequences. Ober (2002) used 28S rDNA and *wingless* (*wg*) to investigate the monophyly and sister group relationships of the carabid subfamily Harpalinae. Maddison et al. (1999) and Ober (2002) have demonstrated strong support for a close relationship of brachinine bombardier beetles and austral psydrines to a monophyletic Harpalinae. Previous studies, however, did not sample enough taxa within Harpalinae to examine specific tribal relationships, focused on examining intergeneric relationships, or have selected taxa on a regional basis (Cryan et al. 2001; Marinez-Navarro *et al*. 2005; Ribera et al. 2005; Ribera et al. 2006; Sasakawa and Kubota 2007). Within Harpalinae, no large-scale modem phylogenetic analysis has been undertaken of the tribes of this diverse subfamily. Such an investigation of phylogenetic relationships is important for understanding the evolution of diversification, chemical and morphological evolution, and evolution of ecological interactions of this large subfamily of beetles.

In this study, molecular data were used to examine the phylogenetic relationships within harpalines. Partial sequences of the nuclear genes 28S rDNA and *wg* were analyzed in broadly sampled taxa of harpaline tribes. Previous work (Ober 2002) was extended by adding new 28S sequences from 134 harpaline beetles and 108 new *wg* sequences. Phylogenetic hypotheses are developed for tribal level relationships among harpalines. The present study has two objectives: first, to examine the phylogenetic relationships within the Harpalinae and to determine the evolutionary relationships and monophyly of many of the tribes; and second, to assess the monophyly of the lebiomorph assemblage (Erwin 1991b; Jeannel 1941) and compare the suggested relationships of tribes with those inferred from molecular sequence data.

## Methods

### DNA extraction, amplification, and sequencing

Genomic DNA samples were prepared from fresh or frozen beetles, beetles preserved in 95–100% EtOH or silica gel following the protocol in Maddison et al. (1999). Voucher specimens have been deposited in the D. Maddison Voucher Collection at the University of Arizona or in the K. Ober Voucher Collection at the College of the Holy Cross. Approximately 1050 base pairs of the D1 – D3 region of 28S rDNA and 500 base pairs of the *wg* gene were amplified and purified for each species with the protocols and primers described in Ober (2002) or with the alternative 5′ *wg* primer 5′ WGB 5′ ACBTGYTGGATGCGNCTKCC-3′. 28S PCR products from *Diploharpus*, *Oxycrepis*, *Teukrus*, *Endyomena*, *Harpalus*, and *Adrimus* and *wingless* PCR products from *Antimerina*, *Atranus*, *Badister reflexus*, *Brachyctis*, and *Incagonum*, were gel-purified and cloned with a TA-Cloning kit from Invitrogen in the pUC 18 plasmid in *Eschenchia coli* INVα using standard protocols and sequenced with the M13R and T7 primers. Sequencing of both strands of the PCR product with the PCR primers for *wingless*, and the PCR primers or modified primers (D1ALT 5′ -AAAGAAACTAACWAGGTT-3′ and D33′ALT 5′-TTCACCATCTTTCGGGTCC-3′) for sequencing 28S was performed by the DNA Sequencing Service, Laboratory of Molecular Systematics and Evolution, at the University of Arizona, using an ABI automated DNA sequencer. Individual sequences were assembled and ambiguous and conflicting bases were corrected using Sequencher 3.0 (Gene Codes Corp.). The sequences generated for this study have been deposited in GenBank, and their accession numbers are listed in Appendix 1.

### Taxon Sampling

Included in this study were members of 34 tribes of Harpalinae (Appendix 1 (#app 1-8-52) ), representing about 85% of the harpaline tribes (Lorenz 2005). 28S sequences were collected from 213 taxa, 193 harpalines, and 20 outgroup taxa. The outgroup taxa represent carabid lineages considered to be closely related to Harpalinae, including brachinine bombardier beetles and austral psydrines, and other more distantly related taxa to connect harpalines to the rest of Carabidae. The *wg* gene fragment could not be amplified or sequenced from some taxa in this study. Therefore the *wg* data set contained 173 taxa, 157 of which are harpaline taxa, and 16 outgroup taxa.

### Alignment

The *wg* gene fragment in this study included the 3′ coding region, *wg* sequences were aligned by eye in MacClade 4.0 (Maddison and Maddison 2000) using the translated amino acid alignment as a guide for the nucleotide alignment. The initial protein alignment was produced by ClustalW 1.7 (Thompson et al. 1997) and adjusted by eye in MacClade with taxon names hidden.

28S rDNA sequences were aligned initially with Clustal W using default settings and subsequently aligned by eye with reference to secondary structure folding models of Gutell et al. (1994) and Gillespie et al. (2004). Taxon names were hidden and sequences were ordered according to the Clustal W guide tree. Regions that could not be aligned unambiguously were excluded from phylogenetic analyses.

ClustalW was used to produce ten different alignments based on the following arbitrary gap opening:gap extension costs: 20:5, 15:3, 12:7,10:5, 10:2, 8:3, 7:2, 5:1, 3:2, and 3:0.05, randomly reordering the taxa for each Clustal alignment (Maddison et al. 1999; Ober 2002). Each of the ten alignments was judged by their obvious misalignments of nucleotides and the number of artificial columns of single or a few nucleotides surrounded by gaps created by Clustal and the number of hyper-variable regions with taxon names hidden and taxon ordering unfamiliar. The Clustal alignments were ranked, and the best one (28SH1, 15:3) was chosen to be the matrix used in phylogenetic inference.

The aligned *wg* data and 28S by eye (28Sbe) alignment were combined into a single a single data matrix, as were the 28S H1 alignment and the *wg* alignment. All 213 taxa were included even though 40 taxa were missing *wg* data. The same regions excluded in the separate 28S data sets were excluded in the combined data sets. Nexus files for all data sets are available from K. Ober.

### Phylogenetic Analyses

Maximum parsimony (MP), maximum likelihood (ML) using PAUP* 4.0vb10 (Swofford 2003), and Bayesian Inference using MrBayes v3.1 (Ronquist and Huelsenbeck 2003) were carried out on the *wg* dataset, the 28Sbe data set, and the combined 28Sbe+*wg* data set. Strong molecular evidence from 18S rDNA (Maddison et al., 1999; Ober 2002), 28S rDNA, and *wg* (Ober 2002) indicates that the subfamily Harpalinae (*sensu*Erwim 1985) is monophyletic, therefore all results of analyses presented in this study have Harpalinae constrained to be monophyletic. If Harpalinae is not constrained to be monophyletic, brachinines move within the subfamily in some analyses.

Phylogenetic trees were reconstructed using MP heuristic searches for all data sets (*wg*, 28Sbe, 28SH1, combined 28Sbe+*wg*, and combined 28SH1+*wg*) using the parsimony ratchet (Nixon 1999), as implemented in PAUPRat (Sikes and Lewis 2001) and PAUP*. The ratchet search was run for 200 iterations for each data set followed by TBR branch swapping. The trees resulting from the ratchet search were submitted to an addition TBR heuristic search in PAUP*. All characters were treated as unordered, and gaps were treated as missing data. Internal support was evaluated by nonparametric bootstrap based on 1000 bootstrap replicates saving only 10 trees per replicate using TBR branch swapping and simple stepwise addition. Robustness for clades of particular interest was evaluated using decay scores (Bremer 1988, 1994) using PAUP* and a command file from MacClade 4.0.

Phylogenetic trees for *wg*, 28Sbe, and 28Sbe+*wg* data sets were estimated using ML methods as implemented in PAUP*. Analyses were performed according to the General Time Reversible substitution model with among-site rate variation (i.e., GTR+I+*Γ*). For all data sets model selection was determined by likelihood ratio tests with starting trees obtained from a simple likelihood search as described in Ober (2002). Searches for highest likelihood trees, with fixed parameter values, consisted of SPR branch swapping and two random addition sequence replicates. (See Appendix 2 for ML search parameter values.)

Finally, trees were inferred for the *wg*, 28Sbe, and the 28Sbe+*wg* data sets using MrBayes. Four independent Bayesian runs were performed for each data set using Metropolis-coupled Markov chain Monte Carlo (MCMCMC) simulations run for two million generations for the 28Sbe+*wg* data, three million generations for the *wg* data, and ten million generations for the 28Sbe data, using four simultaneous MCMCMC chains. Trees were sampled every 100 generations from the approximated posterior distribution. The GTR+I+*Γ* parameters of each process partition (28S partition and *wg* codon positions) were set to be independent and were estimated using Bayesian analysis. Prior distributions for all parameters were set to MrBayes defaults. Bayesian topology and branch posterior probabilities were computed by majority rule consensus after removing all “pre-burnin” trees (Mau et al. 1999). Burnin was determined by plotting likelihood scores against generation number discarding samples before the likelihood score has reached stationarity, approximately the first 30% of all samples.

### Hypothesis testing

Additional MP analyses were performed to specifically address several tribal relationships within Harpalinae. MPTs were examined for each data set using the parsimony ratchet as above. In each search, one of the following was constrained to be monophyletic within Harpalinae: lebiomorph assemblage [Lebiini (including *Celeanephes* and excluding *Plagiotelum*), Cyclosomini, Graphipterini, Catapiesini, Odacanthini, Lachnophorini, Pentagonicini, Calophaenini, and Perigonini]; Lebiini (*sensu stricto*); Odacanthitae (Odacanthini, Lachnophorini, Calophaenini, and Pentagonicini); and Graphipterini + Cyclosomini.

**Table 1.**
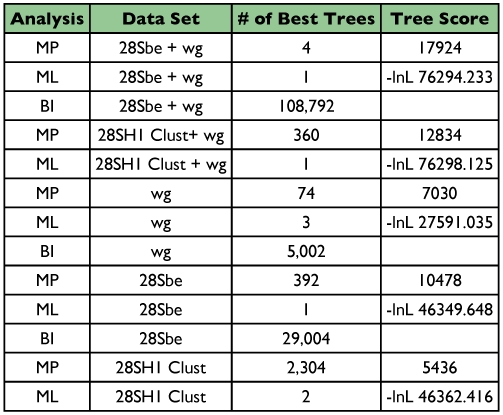
Summary of analyses and results for each molecular data set and analysis method.

## Results

The aligned sequence data consisted of 1233 bases (after excluding 537 ambiguous sites of the alignment) for 28Sbe, 831 bases for 28SH1, 544 bases for *wg*, 1777 bases for 28Sbe+*wg*, and 1375 bases for 28SH1+*wg*. Table 1 shows the summary of phylogenetic analysis results for each data set.

### Combined 28Sbe+*wg*

**Figure 2.**
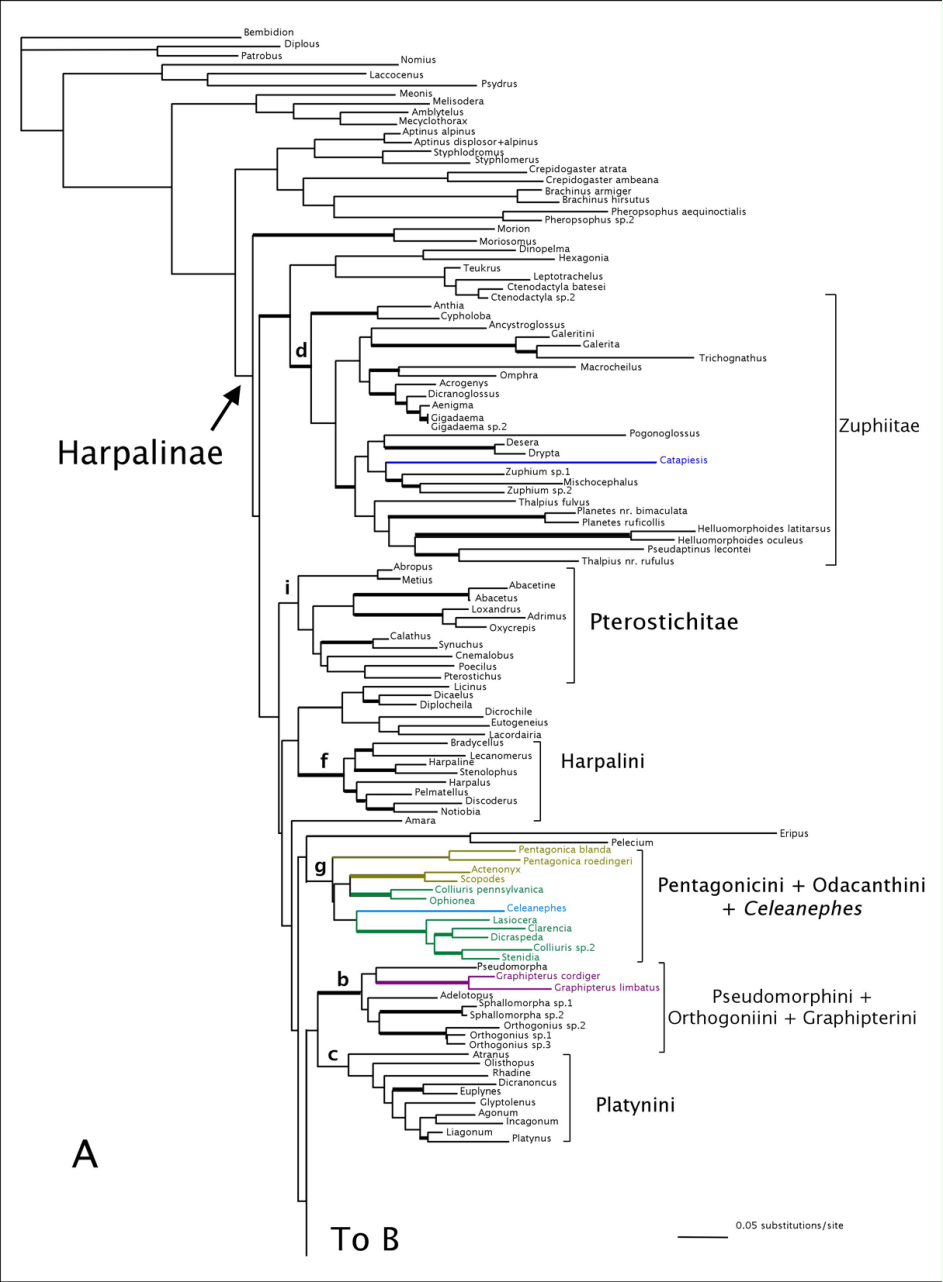
Tree of highest likelihood from the 28Sbe+wg dataset. This tree represents the preferred phylogenetic hypothesis of Harpalinae. Thick branches show clades supported by greater than 95% posterior probability from Bayesian inference. Colored branches are members of the lebiomorph assemblage color coded by tribe. Clade (a) represents Chlaenini + Panagaeini + Oodini group, (b) Orthogoniini + Graphipterini + Pseudomorphini clade, (c) Platynini, (d) Zuphiitae clade, (e) Lebiini, (f) Harpalini, (g) Pentagonicini +Odacanthini clade, (h) Lachnophorini + *Calophaena*, (i) clade comprised of pterostichines and relatives.

MP analyses resulted in four most parsimonious trees (MPTs). A strict consensus of these trees is shown in Figure 1. Bootstrap support for deeper branches and larger clades was low. The results of the ML analyses differed in the deeper relationships among the clades (Figure 2) with low Bayesian posterior probabilities on most deep branches, but the MP and ML trees shared some well-supported similarities (high bootstrap and posterior probabilities) including a monophyletic Zuphiitae (comprised of tribes Anthiini, Dryptini, Galeritini, Helluonini, Physocrotaphini, and Zuphiini) plus Ctenodactylini and Hexagoniini, monophyletic Platynini, monophyletic Harpalini, a clade including pterostichines (plus *Calathus* and *Synuchus*), metiines, abacetines, and loxandrines, and a clade comprised of Graphipterini, Pseudomorphini, and Orthogoniini. Morionines (plus *Badister* in the MP trees) and the Zuphiitae + Ctenodactylini + Hexagoniini occupied positions sister to the rest of harpalines. The large, diverse tribe of Lebiini was largely monophyletic in both the MP and ML trees (Cyclosomini and *Plagiotelum* were included). A common, but not well-supported relationship was seen among the tribes Oodini, Chlaenini, and Panagaeini. This clade included Perigonini in the MP consensus tree.

The results of ML phylogenetic analyses of combined 28SH1+*wg* Clustal alignment were similar to the 28Sbe+*wg* ML tree in overall topology. The results of the *wg*+28SH1 MP analyses (not shown) had few overall deep relationships similar to the 28Sbe+*wg* MP tree. In the MP and ML trees, the tribes Platynini, Lachnophorini, and Harpalini were monophyletic. Abacetines and loxandrines formed a clade, as did Graphipterini + Pseudomorphini + Orthogoniini. The ML tree had a Zuphiitae clade together with ctenodactylines + hexagoniines, however in the MP tree, some galeritines were not included in the clade.

### wingless

**Figure 3.**
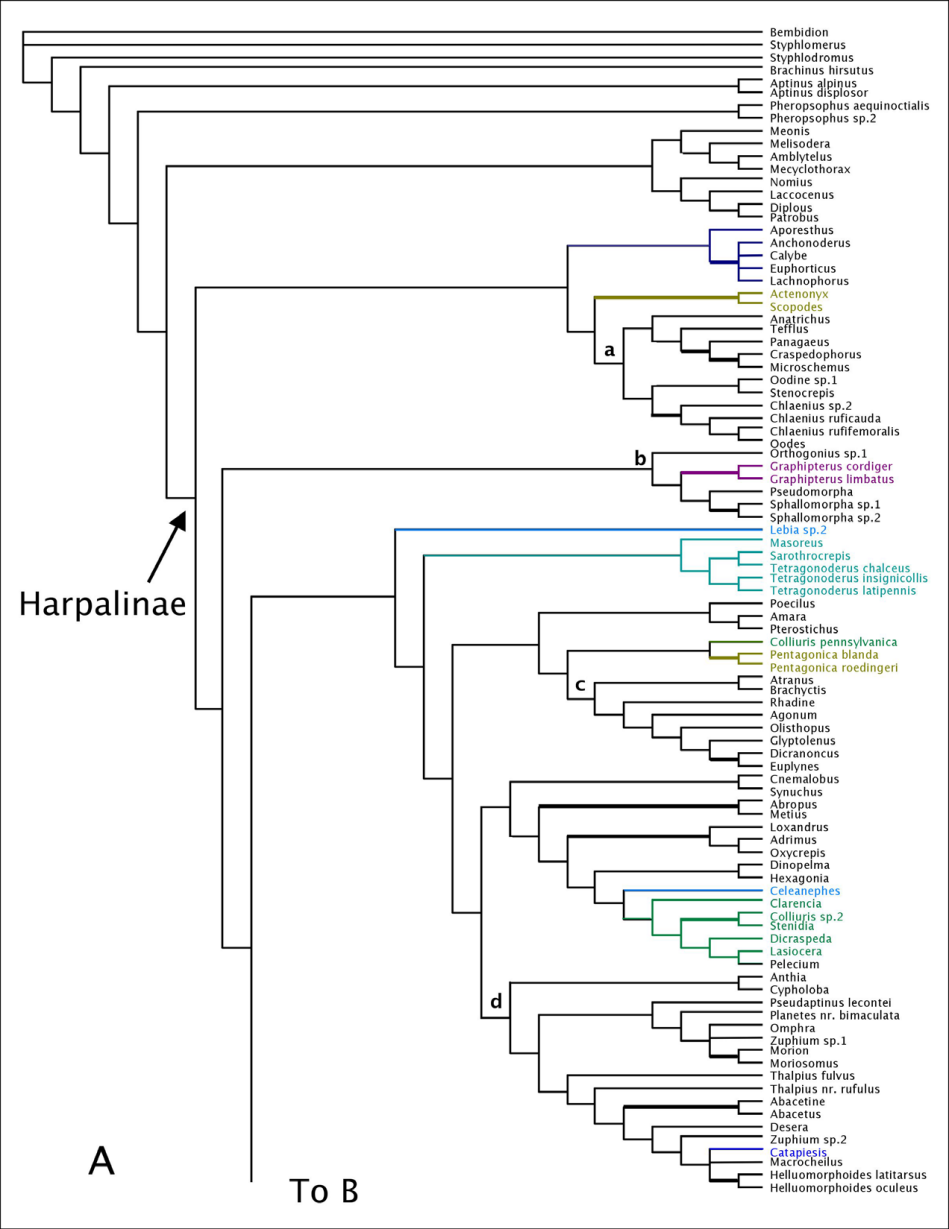
Strict consensus tree of 74 most parsimonious reconstructions of *wg* dataset. Thick branches show clades supported by greater than 70% bootstrap. Colored branches are members of the lebiomorph assembledge color coded by tribe. Clade (a) represents Chlaenini + Panagaeini + Oodini group, (b) Orthogoniini + Graphipterini + Pseudomorphini clade, (c) Platynini, (d) Zuphiitae clade, (e) Lebiini, (f) Harpalini.

Figure 3 shows the strict consensus of 74 MPTs. The deeper branches of the *wg* harpaline tree and most clades have little bootstrap support. Most of the bootstrap support was for clades at the tips of the tree. Lachnophorines, and a clade containing oodines, chlaeniines, and panagaeines were sister to the rest of Harpalinae. The majority of the Zuphitae clade was paraphyletic with respect to Morionini and *Catapiesis*. *Galerita* plus ctenodactylines were found in a group with lebiines. Orthogoniines, pseudomorphines, and graphipterines formed a clade. Platynini and Harpalini were each monophyletic, but many other tribes were not monophyletic. Lebiines were not monophyletic and pieces of this tribe were grouped with Harpalini, Licinini (in part), *Eripus*, *Caelostomus*, and Perigonini. Cyclosomines were not associated with the lebiine clade in this tree.

**Figure 4.**
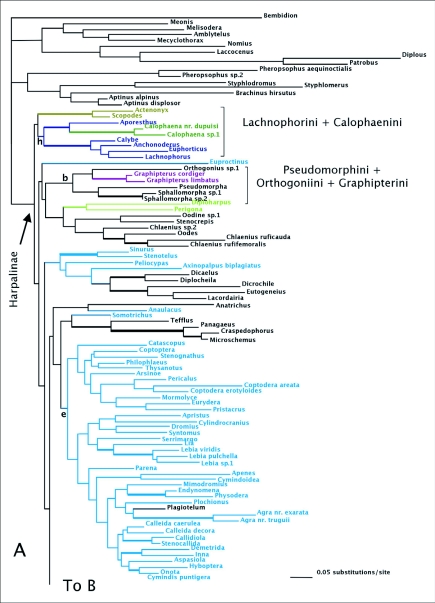
One of three trees of highest likelihood from the *wg* dataset. Thick branches show clades supported by greater than 95% posterior probability from Bayesian inference. Trees differ un the relationships of immediate descendants of the node marked by *. Colored branches are members of the lebiomorph assemblage color coded by tribe, (b) represents Orthogoniini + Graphipterini + Pseudomorphini clade, (c) Platynini, (e) Lebiini, (f) Harpalini, (h) Lachnophorini + *Calophaena*.

ML analyses found 3 trees of high likelihood (Figure 4). Like the MPTs, many tribes were polyphyletic. Platynines and harpalines were each monophyletic, and orthogoniines, pseudomorphines, and graphipterines formed a clade. Zuphiitae was not monophyletic and some tribes were associated with other taxa including *Celeanephes*, *Catapiesis*, and morionines among others. Cyclosomines were not associated with the lebiine clade.

### 28S rDNA

MP analyses of the 28Sbe data set resulted in 392 MPTs in two islands (island 1 of 384 MPTs and island 2 of 8 MPTs). A strict consensus of all 392 MPTs (not shown) suggested little resolution at deeper, tribal level relationships although most tribes were monophyletic but without strong bootstrap support. Pseudomorphines + orthogoniines + graphipterines formed a clade with low bootstrap support. In all MPTs, *Synuchus* and *Calathus* were grouped with pterostichines, and metiines were the sister group to the rest of Harpalinae. Zuphiitae was monophyletic, with moderate bootstrap support, and sister to the ctenodactylines. Lebiines were polyphyletic. One island of MPTs had Licinini (in part) branching after Metiini at the base of Harpalinae. *Celeanephes* + *Amara* were in a clade with odacanthines + pentagonicines and the ctenodactylines + Zuphiitae. The second island of MPTs had *Amara* branching after Metiini at the base of harpalines then a clade of odacanthines + pentagonicines, *Celeanephes*, and pseudomorphines + graphipterines + orthogoniines.

**Figure 5.**
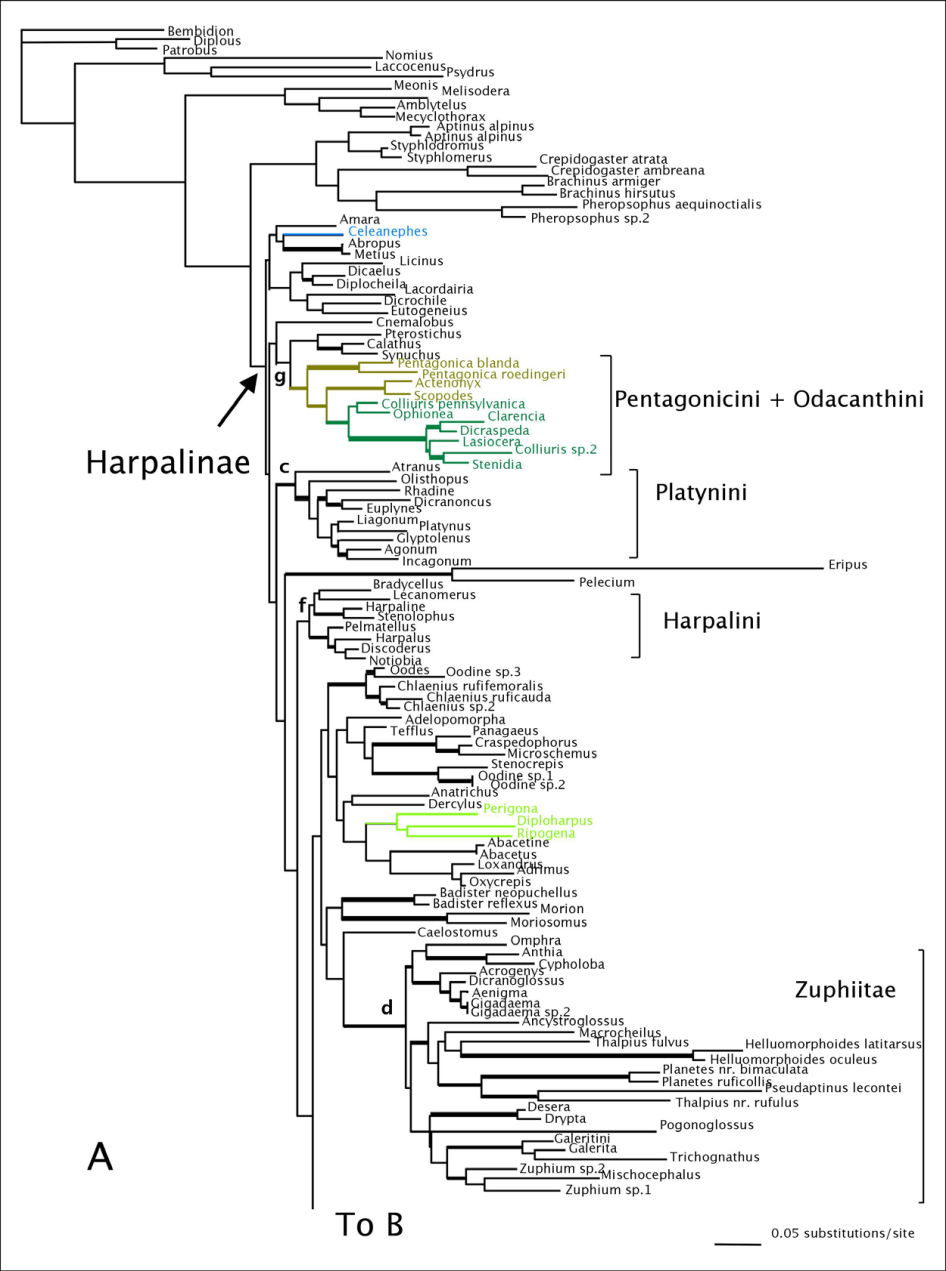
Tree of highest likelihood from the 28Sbe dataset. Thick branches show clades supported by greater than 95% posterior probability from Bayesian inference. Colored branches are members of the lebiomorph assemblage color coded by tribe, (b) represents Orthogoniini + Graphipterini + Pseudomorphini clade, (c) Platynini, (d) Zuphiitae clade, (e) Lebiini, (f) Harpalini, (g) Pentagonicini +Odacanthini clade, (h) Lachnophorini + *Calophaena*.

The ML analyses of the 28Sbe data set resulted in a single tree (Figure 5). The Bayesian Inference yielded little support for most clades especially deeper braches at the base of Harpalinae (Figure 5). However, most tribes were monophyletic and many tribal relationships were similar to results of other analyses. A clade of *Amara*, *Celeanephes*, metiines, and licinines formed the sister group to the rest of Harpalinae. Zuphiite tribes formed a clade near Licinini (in part). Pseudomorphines + graphipterines + orthogoniines formed a clade closely related to Lachnophorini and *Calophaena*. Oodines, chlaeniines, and panagaeines are grouped together. Lebiini (plus Cyclosomini and *Plagiotelum*) was one large paraphyletic group that included ctenodactylines and *Catapiesis*.

Results of the ML and MP analyses of the 28SH1 Clustal alignment (not shown) did not share many similarities with other analyses. Most tribes were not monophyletic in the MPTs. The 2304 MPTs showed a Zuphiitae clade, a monophyletic Platynini, a clade with pseudomorphines + orthogoniines, and *Celeanephes* with Lachnophorini. The two best ML trees had most tribes monophyletic and shared many clades in common with the 28Sbe analyses and other data sets, including a monophyletic Zuphitae, monophyletic Pseudomorphini + Graphipterini + Orthogoniini, and Odacanthini + Pentagonicini.

### Summary of phylogenetic results

In general, there was very little consensus for the deeper relationships within Harpalinae from 28S rDNA and *wg* data. Results of most analyses conflicted on the intertribal relationships, and there was little or no support for these deep clades. Phylogenetic relationships conflicted among trees from different analyses. Thus, these data sets are sensitive to the optimality criterion and alignment method. However from these analyses a few general similarities can be observed. The lebiomorph assemblage was polyphyletic and the tribes comprising this group were scattered throughout the tree in four to nine groups depending on dataset and analysis. The tribe Lebiini was largely monophyletic or split into a few clades, and included the tribe Cyclosomini and the genus *Plagiotelum*. *Celeanephes* was not grouped with other members of Lebiini, but its position was variable. Odacanthines and lachnophorines do not form a clade, instead pentagonicines were the sister group to odacanthines and *Calophaena* was the sister group to lachnophorines.

**Figure I.**
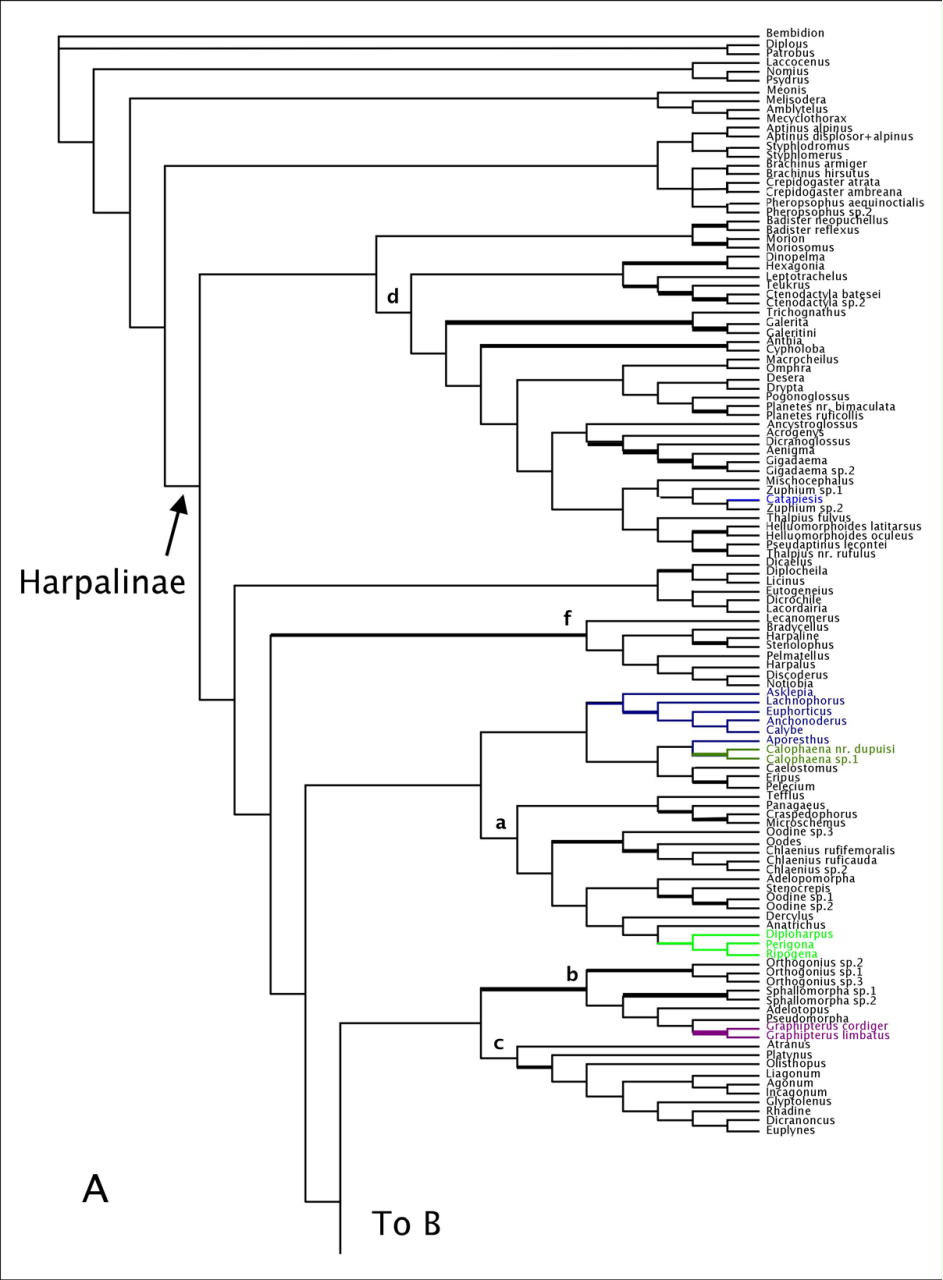
Strict consensus tree of four most parsimonious reconstructions of combined 28Sbe+*wg* dataset. Thick branches show clades supported by greater than 70% bootstrap. Colored branches are members of the lebiomorph assemblage color coded by tribe. Clade (a) represents Chlaenini + Panagaeini + Oodini group, (b) Orthogoniini + Graphipterini + Pseudomorphini clade, (c) Platynini, (d) Zuphiitae clade, (e) Lebiini, (f) Harpalini, (g) Pentagonicini +Odacanthini clade, (i) clade comprised of pterostichines and relatives.

**(con't).**
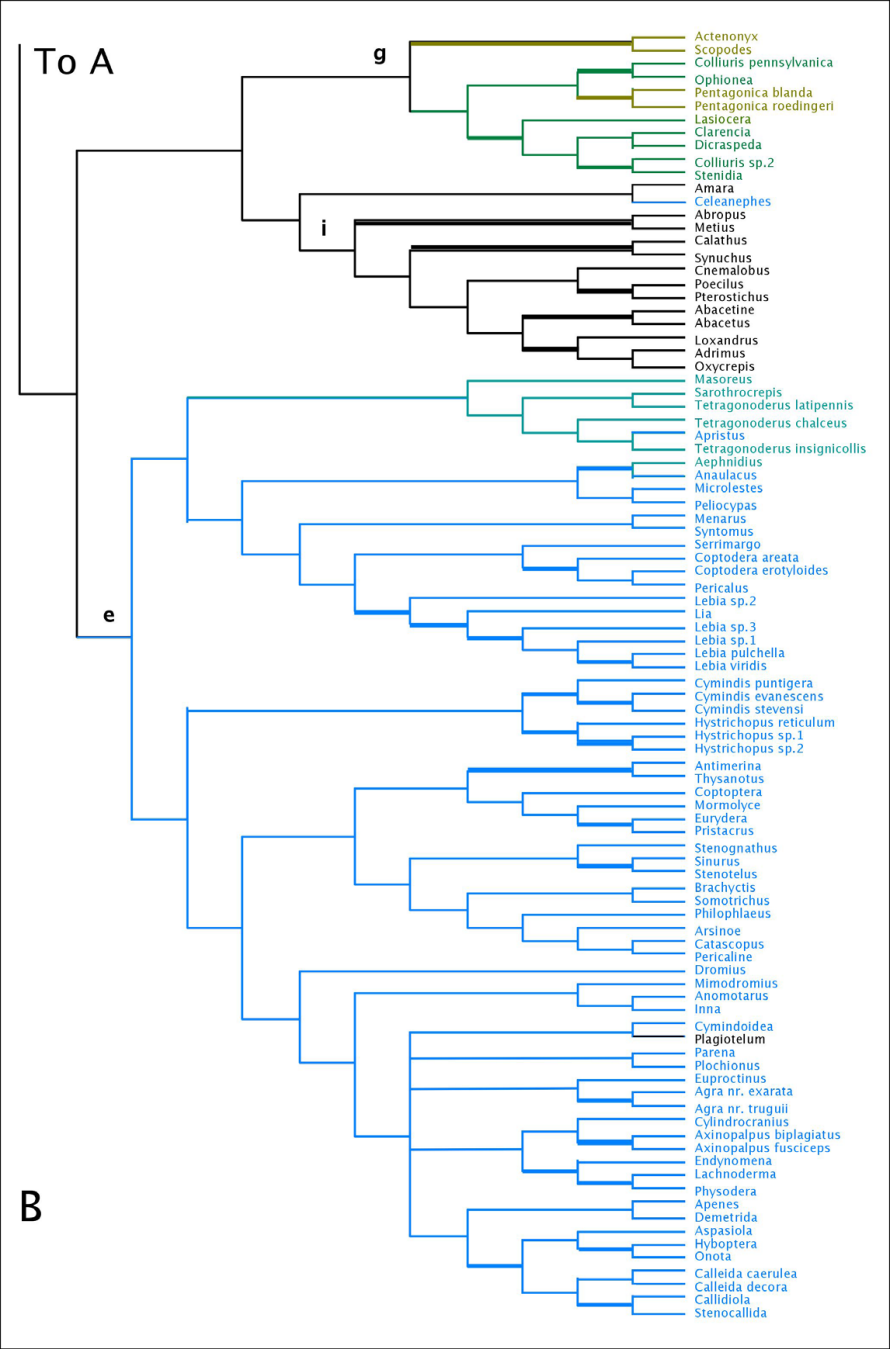

**(con't).**
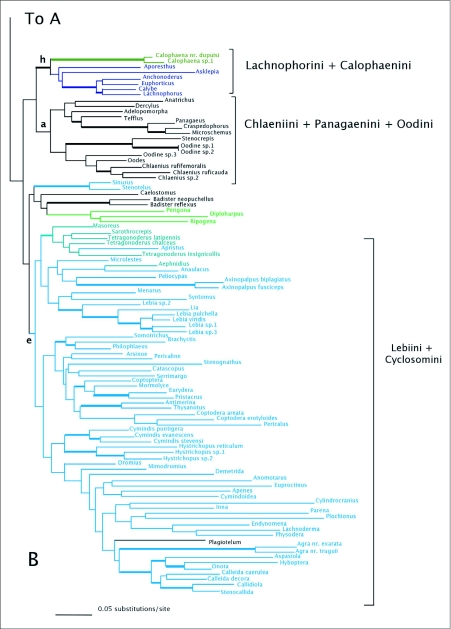

**(con't).**
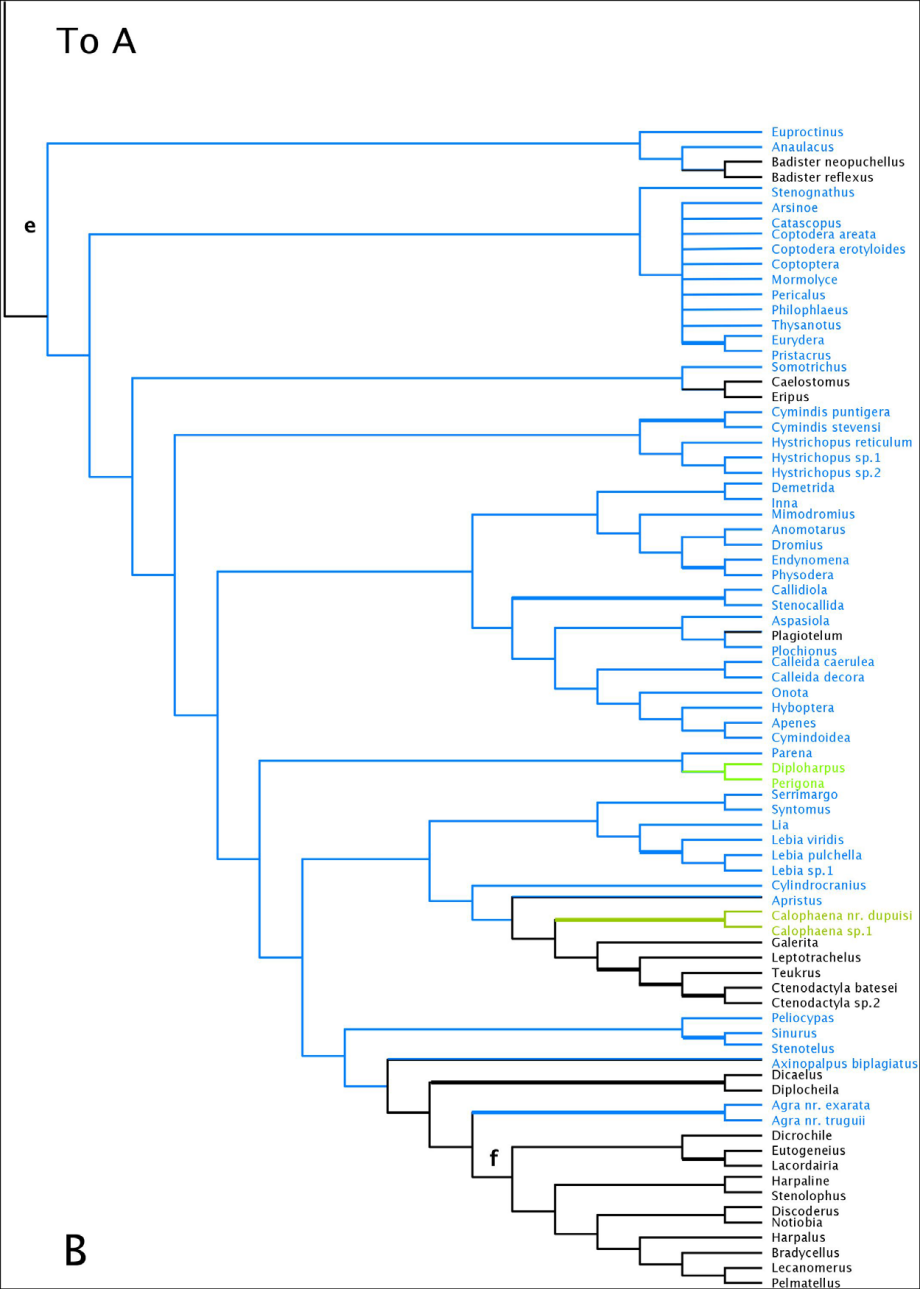

**(con't).**
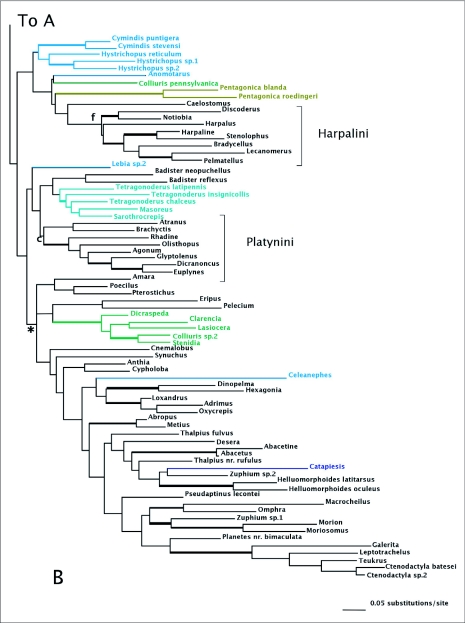

**(con't).**
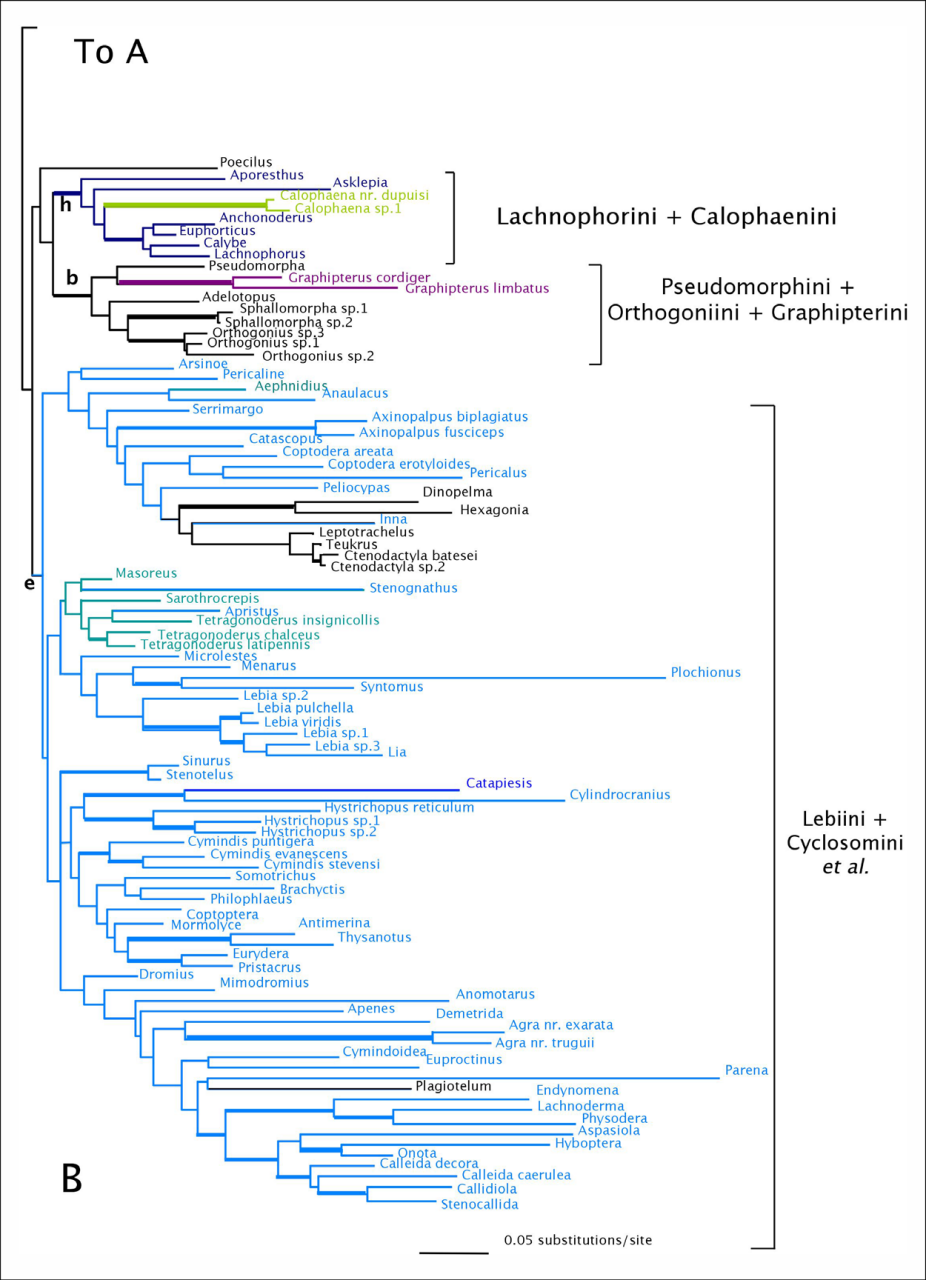

There were some clades that were found in most or all trees, and many include relationships congruent with current classification and morphological studies. Besides the well-supported monophyly of the tribes Platynini and Harpalini, few other clades were well supported. *Calathus* and *Synuchus* were more closely related to pterostichines than platynines. An unexpected clade containing Pseudomorphini + Graphipterini + Orthogoniini was strongly supported (high bootstrap values and posterior probabilities) in nearly all trees. A large Zuphiitae clade containing the tribes Anthiini, Dryptini, Galeritini, Helluonini, Physocrotaphini, and Zuphiini was also well supported in most analyses although some of the tribes within this large clade were not monophyletic. Figure 2 shows the preferred phylogenetic tree of Harpalinae based on the combined 28Sbe+*wg* data set. The results of the phylogenetic analyses in this study are summarized in Tables 1 and 2.

### Hypothesis testing

Table 2 summarizes the results of tests for phylogenetic hypotheses of several tribal relationships. The analyses reflected several relationships that have been previously suggest from morphological studies. In all cases, there was no evidence for a monophyletic lebiomorph assemblage containing Lebiini (including *Celeanephes* and excluding *Plagiotelum*), Cyclosomini, Graphipterini, Catapiesini, Odacanthini, Lachnophorini, Pentagonicini, Calophaenini, and Perigonini in a single clade. Likewise there seems to be no evidence to support alternative hypotheses including a strictly monophyletic Lebiini (including *Celeanephes* and excluding Cyclosomini and *Plagiotelum*), a sister group relationship between Graphipterini and Cyclosomini, and a clade consisting of Odacanthini + Lachnophorini + Calophaenini + Pentagonicini.

## Discussion

Despite little support for the deeper phylogenetic relationships of Harpalinae, a problem encountered in previous studies (Ober 2002), many of the smaller clades of harpalines were resolved and well supported from 28S and *wg* data (Figure 2). Some conclusions about harpaline relationships can be reached (see below). Tribal affiliations of genera were largely concordant with previously proposed groupings based on morphological traits and were generally well supported. The relationships of a number of tribes and genera are still enigmatic, and may only be resolved by the inclusion of additional data and denser taxon sampling.

### Relationships of particular harpaline taxa

Phylogenetic relationships of many lineages within Harpalinae remain unresolved. It is beyond the scope of these analyses to address all of the phylogenetic issues. It appears that 28S and *wg* data alone are not sufficient to clearly resolve the relationships among many of these controversial groups, so we have chosen to highlight a few groups whose relationships are particularly interesting in light of the morphological and molecular data.

### Lebiomorph Assemblage

The lebiomorph assemblage is a group of harpaline tribes whose members possess an unusual defensive chemical delivery system: the opening of the ducts that secrete defensive chemicals are located at the eighth abdominal tergite and are shaped like turrets (Deuve 1993; Erwin 1985; Erwin in litt; Forsyth 1972), and most possess truncate elytra (probably to accommodate the defensive chemical delivery). The lebiomorph assemblage is more or less Lebiitae (tribes Lebiini, Cyclosomini, Graphipterini, Perigonini, Odacanthini, Lachnophorini, and Pentagonicini) of Erwin (1991b), however we have also included in this group Catapiesini and Calophaenini. Catapiesini, previously included in Lebiitae by Erwin (1984), has truncate elytra and specialized eighth abdominal tergite turrets, although the turret size is smaller than in other lebiomorphs. Calophaenini has recently been included within Lachnophorini (part of the lebiomorph assemblage) by Liebherr (1988) based on a notable synapomorphy from the female reproductive tract. Calophaenines have shortened elytra, but no obvious turreted chemical delivery system, and thus were previously considered to have lost this feature.

A monophyletic lebiomorph assemblage was never recovered in any of our analyses. Even when *Plagiotelum*, *Celeanephes* , and *Catapiesis* were excluded from the lebiomorph assemblage, it is still highly polyphyletic. A polyphyletic lebiomorph assemblage has important implications for the evolution of carabid defensive chemical delivery system. Within harpalines, it appears the eighth abdominal tergite turrets have evolved independently at least four times. This character, previously considered to be the synapomorphy for lebiomorphs, is apparently homoplasious and similarities may be due to convergent evolution. It is clear that future work must include a detailed morphological study of these turrets in lebiomorph and other harpaline taxa to determine their structure, function, and evolutionary history.

### Odacanthitae

Within the lebiomorph assemblage, Liebherr (1988) described the Odacanthitae, a supertribe united based on a synapomorphy from the female reproductive tract; members of the tribes Lachnophorini, Calophaenini, Odacanthini, and Pentagonicini share a bipartite spermatheca (Leibherr 1988). Results of the molecular analyses did not support the monophyly of Odacanthitae (0.2% of Bayesian trees had a monophyletic Odacanthitae). However, most analyses showed a close relationship between Pentagonicini and Odacanthini (except in trees from the *wg* dataset). Liebherr (1988) combined the pentagonicines *Scopodes*, *Actenonyx*, and *Pentagonica* within Odacanthini, and this combination was supported, for the most part, by the molecular data. *Actenonyx* has sometimes been placed in the Lebiini based on mouthparts and suborbital setae (Ball, Kavanaugh & Moore, 1995s). Britton (1940) also originally placed *Actenonyx* with lebiines, but later Britton (1941) suggested that the genus was more closely allied with the odacanthines. No analysis placed *Actenonyx* with a lebiine taxon, instead it was sister to *Scopodes* usually within the Pentagonicini + Odacanthini clade. The results from most of the analyses place *Calophaena* (Calophaenini) near or within Lachnophorini (except in the *wg* parsimony tree). This is in agreement with morphological analyses by Leibherr (1988). Despite the affinity of *Calophaena* with Lachnophorini and Pentagonicini with Odacanthini, these two clades are not sister groups and the bipartite spermatheca may have evolved multiple times as suggested by its presence in other unrelated taxa such as *Glyptolenus* (Liebherr 1988).

**Table 2.**
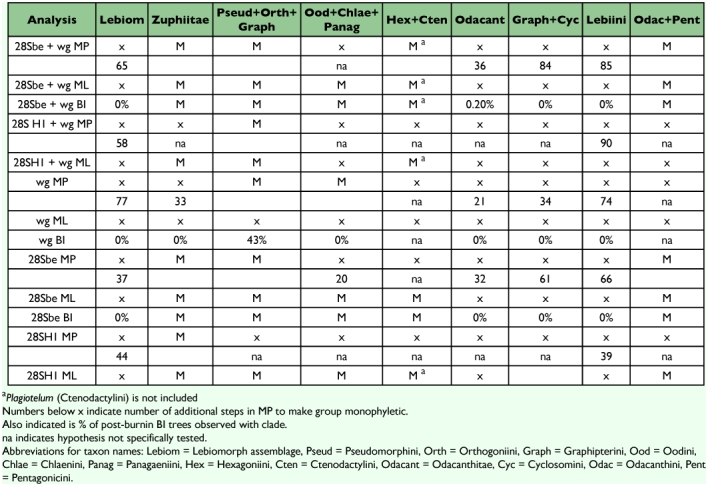
Status of selected taxa in results of phylogenetic analyses.

### Cyclosomini + Graphipterini

Some morphological classifications have placed two lebiomorph tribes, graphipterines and cyclosomines, in close association. Chaudoir (1876) recorded that adults of Graphipterides (Graphipterini) exhibited modified tibial spurs common to members of the cyclosomines. Jedlicka (1963) also recognized a relationship between graphipterines and cyclosomines (or groups included in or near cyclosomines). Jeannel (1949) included cyclosomines and graphipterines within his “family Masoreidae.” Kryzhanovsky (1976) treated Masoreomorphi as a supertribe that included Masoreini (Cyclosomini) and Graphipterini. In most trees from the analyses (except *wg* trees), cyclosomines tended to be associated with members of dromiine and/or cymindine lebiines and never form the sister group of graphipterines. Along with the tibial spur character, cyclosomines and graphipterines also exhibit various sorts of reductions of stylomere two of the female ovipositor. While graphipterines and cyclosomines share reductions in stylomere two, Dromiina lebiines also exhibit the same sort of stylomere reductions (Ball 1982; Habu 1967).

The inclusion of cyclosomines within lebiines was unexpected but not surprising. Cyclosomines, almost all xeric, ground-dwelling beetles except for the arboreal *Sarothrocrepis*, often appeared in the phylogenies near xeric, ground-dwelling lebiines (dromiines and cymindines). Shaum (1860) recognized that *Masoreus* and *Tetragonoderus* were related to Lebiini, and Csiki (1932) placed members of Lebiini and Cyclosomini next to each other, implying a close relationship among these taxa. Cyclosomines were not always monophyletic in the results of separate and combined analyses. *Anaulacus* was not associated with other cyclosomines and may represent an independent lineage not closely related to the rest of the tribe (Ball and Shpeley 2002).

### Lebiini

Lebiini has more than 4200 species (Lorenz 2005) and is represented in all major zoogeographical regions of the world, but is especially diverse in the tropics. Lebiines are strikingly divergent in form, color, and in natural history, making it difficult to provide a clear synapomorphy for the tribe. No overall phylogeny of Lebiini exists, and most efforts of systematists have focused within subtribes. Ball (1975) suggested that subtribes Pericalina and Apenina are primitive, based on the ovipositor, forming the basal stock of lebiines that evolved from a platynine-like ancestor. Cymindines are more evolutionarily intermediate with respect to Calleidina and Lebiina. Phylogenetic analysis of lebiine subtribes based on morphological characters by Ball et al (1995) agreed with these previous ideas of lebiine subtribal relationships. Ball (1975) also suggested that lebiines may be paraphyletic due to the great diversity of form and habitats within the tribe and lack of many clear synapomorphies. Based on female ovipositor characters and characters from the mentum, Basilewsky (1984) seemed to imply a polyphyletic Lebiini from his schematic of the phylogeny of Lebiinae which has many lineages of lebiines placed as paraphyletic with respect to other unidentified harpaline lineages and other tribes such as zuphiines and cyclosomines sharing a close relationship with some lebiines.

Results of analyses in this study indicate that Lebiini (*sensu stricto*) is not monophyletic. Cyclosomines were included within the group, as was *Plagiotelum*, a genus previously classified as a ctenodactyline because of its elongated prothorax and complete elytra. Additionally, *Celeanephes*, an enigmatic genus that has been placed in Lebiini (Ball 1995) was not clearly associated with any lebiine taxa, however its placement varied between analyses. Beyond these nontraditional lebiine taxa the remaining lebiines fell into one or two large lineages, which were sometimes paraphyletic. Our results support a small number of clades containing most lebiines, however support for any single phylogenetic arrangement is low and so the relationship of lebiines to other harpaline tribes remains unclear.

Lebiine clades found in the analyses do not correspond to subtribes, and subtribes were never recovered as monophyletic. However, the classification of lebiine subtribes has been unstable (Ball 1975; Ball and Hilchie 1983; Ball and Shpeley 1983; Ball et al. 1995; Casale 1998; 430 Jeannel 1949; Shpeley 1986), and subtribal boundaries and composition are difficult to determine, but there is no clear correspondence with these previous hypotheses. A group of lebiines, including some traditionally classified in the subtribe Calleidina, appears in most phylogenies and is largely concordant with the calleidine phylogeny of Casale (1998) (e.g. *Physodera* , *Agra* , *Anomotarus* , *Qnota* , *Plochoinus* , *Demetrida* , *Mimodromius* , Metallicina, and the “Callides” of Chaudoir (1872)) based mainly on reproductive tract characters from males and females. At present, lebiine phylogenetic relationships remain unresolved and problematic.

### Zuphiitae

The well-supported zuphiite clade (comprised of tribes Anthiini, Dryptini, Galeritini, Helluonini, Physocrotaphini, and Zuphiini) was found in most trees (paraphyletic in *wg* trees). However, the tribes Galeritini, Helluonini, and Zuphiini within Zuphiitae were not monophyletic and more taxon sampling needs to be done to explore the tribal boundaries within Zuphiitae. The longest branches within the harpaline clade were found in Zuphiitae. Long branch attraction (Felsenstein 1978) may be one reason for the zuphiite grade seen at the base of the *wingless* parsimony tree or a paraphyletic Zuphiitae seen in the 28S parsimony tree, the *wingless* distance and ML trees, and the 28S+*wingless* combined distance tree, where other long branches such as *Eripus* and *Catapiesis* are sometimes found with zuphiite taxa. In most trees, the Zuphiitae can be found in a basal clade in harpalines.

The six tribes in Zuphiitae were proposed to form a single clade by Basilewsky (1984) based on antennal characters and spination on the first stylomere of the female ovipositor. Jeannel (1949) placed together zuphiines, dryptines, and galeritines in Dryptidae and followed them immediately by Anthiidae (anthiines and helluonines). Erwin (1985) described a similar arrangement. From this, one can infer that these authors regarded the dryptite and anthiite lineages each as monophyletic and the supertribes themselves fairly closely related, but not forming a monophyletic group.

### Pseudomorphines + Orthogoniines + Graphipterines

There was an unexpected but strongly supported clade of orthogoniines, graphipterines, and a paraphyletic Pseudomorphini. No obvious morphological synapomorphies link these three tribes. Pseudomorphines have a very unusual ovoid adult body form along with other highly autapomorphic structures and larval habits, due in part to their association with ants (Erwin 1981; Moore 1974). While they are clearly harpalines (Maddison et al. 1999; Ober 2002), they have been considered a distinct lineage, in some classifications receiving the rank of subfamily (Lindroth 1969) or family (Notman 1925). Evidence from paramere vestiture and basal bulb of the male median lobe on the aedeagus, male tarsal setae (Erwin 1981) suggests that pseudomorphines are related to pterostichines in Harpalinae, and adult chemical defense of formic acid and hydrocarbons (Moore 1979; Moore and Wallbank 1968) places pseudomorphines among the members of Harpalinae. Orthogoniines have never been suggested to be related to either pseudomorphines or graphipterines. They were associated with licinines by Jeannel (1948), to several tribes of the Zuphiitae and lebiines by Kryzhanovsky (1976), and to idiomorphines, catapiesines, and amorphomerines by Erwin (1985; 1991a). Graphipterines were thought to be closely related to cyclosomines as both tribes share a modified hind tibial spur (see above).

While morphology does not support a close relationship among these three tribes, all or some of their members have obligate relationships with social insects. Orthogoniine larvae are obligate symbionts of termite nests (Bousquet and Larochelle 1993; Erwin 1979; Kistner 1982; Wasmann 1902) and prey on termites, and some orthogoniine adults also live in termite nests (Kistner 1982). Pseudomorphines are myrmecophilous and have highly modified morphological structures in adults and larvae for life with ants (Erwin 1981; Moore 1974). Pseudomorphine larvae eat adult and larval ants (Lenko 1972; Moore 1974). Larvae of some species of *Graphipterus* are obligate symbionts in ant nests and prey on ant eggs and larvae as a specialized predator or parasitoid (Brandymayr *et al*. 1994; Paarman 1985). The larvae of all three tribes are physogastric in the last two instars (Erwin 1981; Kistner 1982; Paarman 1985), a condition common to parasitic carabids or inquilines of social insects. It seems likely that relationship with social insects evolved early in the shared evolutionary history of pseudomorphines, orthogoniines, and graphipterines. Future work is required to identify morphological synapomorphies, if they exist, for this surprising clade and investigate its evolutionary history.

### Oodini + Chlaenini + Panagaeini + Licinini

Several authors suggest a close relationship of chlaeniines, licinines, panagaeines, and oodines (Jeannel 1942; 1949; Lindroth 1969; Thompson 1979). These tribes have been combined into the Panagaeitae (Castelnau and Brullé 1840; Kirby 1817), a super-tribe in the subfamily Harpalinae containing panagaeines, chlaeniines, oodines, licinines and peleciines, on the basis of the constricted neck and triangular, compressed terminal palpomeres. Erwin (1985), Kryzhanovsky (1976), and Moore et al. (1987) also accepted this grouping. Chaudoir (1878) first suggested that oodines are closely related to panagaeines and chlaeniines. Members of these tribes have a metepisternum coadunate with the elytral epipleuron.

Panagaeines and chlaeniines were thought to form a clade based on phenol in secretions from pygidial glands, whereas oodines secrete methacrylic acid (Bousquet 1987b). Some authors (Ball 1960; van Emden 1942; Jakobson 1906; LeConte 1861) combined chlaeniines and oodines into one tribe. Our results indicate there may be a close relationship between chlaeniines and oodines, although neither tribe was always monophyletic. In most trees, oodines, chlaenines, panagaeines, and *Dercylus* were found together in the same clade (in some cases paraphyletic with respect to Perigonini). Licinines and peleciines were not closely related to chlaeniines, oodines, and panagaeines.

### Conclusions and future work

Our investigation is the largest molecular study on the phylogenetic relationships of the speciose carabid subfamily Harpalinae, and is another step in the progress toward understanding the evolution of this diverse group. This study revealed new monophyletic groups that were robust to different phylogenetic analysis method implemented here. For example, galeritines, anthiines, dryptines, helluonines, and zuphiines were confidently placed together in a single clade, and cyclosomine taxa were placed within Lebiini. Also, the monophyly of Pseudomorphini + Orthogoniini + Graphipterini was established for the first time. Nonetheless several taxa included in this study remain difficult to place (*Celeanephes*, *Caelostomus*, *Calophaena*, *Catapiesis*, *Badister*, Peleciini, *etc*.) with any confidence. These enigmatic taxa were not consistently associated with any clade across data sets and analysis methods. It is imperative that future studies include denser taxon sampling, more sequence data, and additional analyses in order to determine the relationships of these taxa.

Most basal tribal relationships of Harpalinae were not sufficiently clarified in any of the analyses, suggesting that the molecular data in this study do not provide enough information to detect relationships at this level. Such problems may be alleviated by increasing the number of characters, under the assumption that the nucleotides follow the same evolutionary patterns (Berbee et al. 2000). Data from additional genes are needed to increase the support for deeper nodes. Alternatively, if the observed lack of phylogenetic resolution reflects a rapid harpaline radiation, additional data may not improve statistical support at deeper nodes. Short internal branches in the harpaline molecular phylogenies presented in this study and others (Maddison et al. 1999; Ober 2002) indicate that harpalines underwent a rapid radiation at the splitting of the ancestors of what is now roughly the tribal level. The rapid radiation suggested by these molecular data would also explain the paucity of morphological synapomorphies for higher-level tribal relationships in harpalines. The first fossil of a member of the subfamily Harpalinae and its close relatives is from the late Cretaceous, about 90 Myr ago (Ponomarenko 1992). By the Oligocene (approximately 35 Myr ago), species from present day harpaline tribes, even modern genera, are known from Baltic amber (Lindroth 1974). This suggests that the rise of the diversity of present day harpaline tribes was very rapid, and lineage splitting was much faster than accumulation of history-marking changes.

## Figures and Tables

**Appendix 1.**
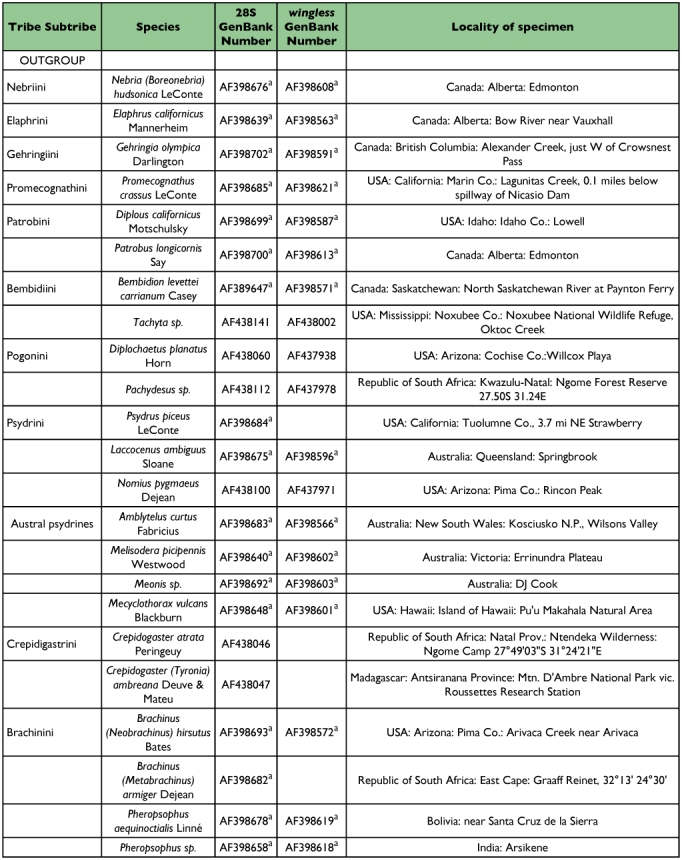
Taxa Sampled

**Appendix 2.**
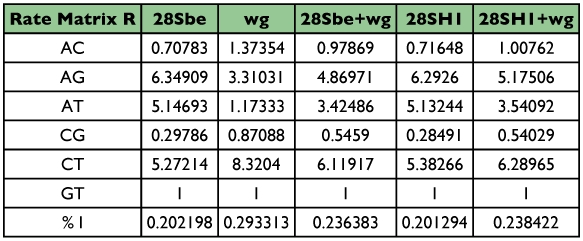
Maximum likelihood analyses rate matrix parameters.

## Abbreviations

ML -: maximum likelihood
MP -: maximum parsimony
MPT -: most parsimonius tree
TBR -: tree bisection reconnection
